# How Driver Oncogenes Shape and Are Shaped by Alternative Splicing Mechanisms in Tumors

**DOI:** 10.3390/cancers15112918

**Published:** 2023-05-26

**Authors:** Weronika Wojtyś, Magdalena Oroń

**Affiliations:** Laboratory of Human Disease Multiomics, Mossakowski Medical Research Institute, Polish Academy of Sciences, Pawinskiego 5, 02-106 Warsaw, Poland

**Keywords:** alternative splicing, mutant p53, KRAS, CMYC, splicing factors

## Abstract

**Simple Summary:**

Alternative pre-mRNA splicing is a process that allows for the generation of an extremely diverse proteome from a much smaller number of genes. In this process, non-coding introns are excised from primary mRNA and coding exons are joined together. Different combinations of exons give rise to alternative versions of a protein. Scientists use RNA sequencing methods to study how alternative splicing is deregulated in tumors. Aberrant splicing affects all features of cancer cells: unlimited growth, avoidance of cell death, invasiveness, angiogenesis, and metabolism. Some tumor driving genes, namely, oncogenes, change alternative splicing by influencing molecular pathways that control it. Alternative splicing can also activate genes and pathways that drive tumor growth. Growing knowledge about deregulation of splicing in cancer helps to design better methods of diagnosis and treatment.

**Abstract:**

The development of RNA sequencing methods has allowed us to study and better understand the landscape of aberrant pre-mRNA splicing in tumors. Altered splicing patterns are observed in many different tumors and affect all hallmarks of cancer: growth signal independence, avoidance of apoptosis, unlimited proliferation, invasiveness, angiogenesis, and metabolism. In this review, we focus on the interplay between driver oncogenes and alternative splicing in cancer. On one hand, oncogenic proteins—mutant p53, CMYC, KRAS, or PI3K—modify the alternative splicing landscape by regulating expression, phosphorylation, and interaction of splicing factors with spliceosome components. Some splicing factors—SRSF1 and hnRNPA1—are also driver oncogenes. At the same time, aberrant splicing activates key oncogenes and oncogenic pathways: p53 oncogenic isoforms, the RAS-RAF-MAPK pathway, the PI3K-mTOR pathway, the EGF and FGF receptor families, and SRSF1 splicing factor. The ultimate goal of cancer research is a better diagnosis and treatment of cancer patients. In the final part of this review, we discuss present therapeutic opportunities and possible directions of further studies aiming to design therapies targeting alternative splicing mechanisms in the context of driver oncogenes.

## 1. Introduction

Alternative pre-mRNA splicing is a common process in higher eukaryotes that allows for the generation of an extremely diverse proteome from a much smaller number of genes [1,2]. It is estimated that 95% of human multiexon genes undergo alternative splicing [3]. Alternative splicing plays a crucial role in development and tissue differentiation by providing proteins with different functions from the same set of genes [4]. Disruption of this process may result in disturbed cell differentiation and oncogenesis. The pattern of alternative exon selection in cancer resembles the one in corresponding embryonic tissues and involves the same regulatory pathways [5]. Multiple mRNA-Seq datasets comparing tumors and corresponding normal tissues demonstrate that an aberrant splicing landscape is a common feature of cancer [6,7,8]. Alternative oncogenic isoforms of different proteins are involved in all hallmarks of cancer: growth signal independence, avoidance of apoptosis, unlimited proliferation, genome instability, motility and invasiveness, angiogenesis, and metabolism [9,10].

mRNA splicing involves the spliceosome (a large ribonucleoprotein complex composed of snRNAs and associated proteins) and splicing regulatory factors [11]. In hematological malignancies, point mutations in spliceosome components (e.g., SF3B1, SF3A1, U2AF1, or ZRSR2) are more frequent, while in solid tumors, splicing is deregulated mostly by upregulation or downregulation of splicing regulatory factors (e.g., SRSF1, TRA2β, hnRNPA1, or QKI) [12]. Splicing is tightly regulated at various levels: by linking with transcription rate [13,14], expression, nonsense-mediated decay, and post-translational modifications of splicing factors [15,16,17]. In addition, relative levels of snRNAs U1, U2, U4, U5, and U6—basic components of the spliceosome—may also be important [18]. All of these can be disrupted by the activation of proto-oncogenes. In this review we will focus on the driver oncogenes and oncogenic pathways that are frequently activated in tumors of different origin, are known to cause phenotypic addiction of cancer cells, and are involved in splicing modification: mutated *TP53*, *CMYC*, *KRAS*, *PI3K*, *SRSF1*, and *hnRNPA1*.

Altered splicing may also increase the activity of driver oncogenes and oncogenic signaling pathways via preferential expression of alternative pro-oncogenic isoforms, creation of new splice sites leading to the expression of cancer-specific isoforms, or indirectly via changed splicing of regulatory proteins. Specific examples will be discussed in the following paragraphs.

Despite decades of studies, key driver oncogenes are still difficult to target therapeutically. Several drugs targeting mutant p53, mutant KRAS, or activated CMYC have been tested in vivo and in clinical trials [19,20,21], but none of them are used in standard therapeutical protocols. Traditional chemotherapy causes serious side effects, while cancer cells easily develop resistance toward single-targeted inhibitors. Therefore, there is a need to search for new cancer vulnerabilities. Aberrant alternative splicing, as a hallmark of all cancers [10], is a good candidate for drug design. The final chapter of this review will discuss currently tested strategies aiming to either precisely correct the splicing of selected genes or to inhibit whole splicing machinery. Researchers in the cancer biology field know that finding effective drug combinations is better than single molecules, because it allows for the prevention of strong side effects and the activation of compensatory resistance mechanisms [22]. Therefore, we need to better understand interdependencies between driver oncogenes and pre-mRNA splicing to select more promising drug combinations.

## 2. Alternative Splicing under the Control of Driver Oncogenes

### 2.1. CMYC

The oncogenic activity of CMYC in different cancers has been extensively studied and well documented. This proto-oncogene belongs to the family of transcription factors, along with NMYC and LMYC [23]. In normal tissues, its level is low and tightly regulated on the transcriptional and post-transcriptional level. In cancers, CMYC level is frequently increased via various mechanisms: gene amplification or translocation, upregulation of signaling pathways boosting CMYC activity, post-translational modifications, and increased protein stability [24,25]. Activated CMYC is a hallmark of many cancers and is required for their initiation and maintenance [26].

The transcriptional program of CMYC and its intersections with other oncogenes have been broadly discussed elsewhere [27,28]. Here, we will focus only on the role of CMYC in the regulation of alternative splicing and its consequences for oncogenesis. The globally increased transcription induced by CMYC requires adaptation of the splicing and translation machinery. The shortage of spliceosome components and splicing regulatory factors affects splicing profile [29,30]. CMYC hyperactivation increases translation of a core spliceosome component, SF3A3, through an eIF3D-dependent mechanism. This affects the splicing of many mRNAs, including those involved in mitochondrial metabolism favoring stem-cell-like, cancer-associated changes [31]. Koh et al. demonstrated that CMYC directly upregulates the transcription of several small nuclear ribonucleoprotein particle assembly genes, including PRMT5, an arginine methyltransferase. This protein methylates Sm proteins that form the Sm core of the spliceosomal snRNPs U1, U2, U4/U6, and U5, and is critical for correct spliceosome assembly [32]. The perturbation in PRMT5 expression caused by CMYC leads mainly to intron retention or exon skipping and disturbs splicing fidelity [30]. CMYC not only causes overexpression of the most studied oncogenic splicing factor, SRSF1 [33], but also causes overexpression of several other proteins from the same family. Urbanski et al. identified an alternative splicing (AS) signature associated with high CMYC activity in breast cancer and showed that the change in AS is caused by the co-expression of splicing factors modules. They also demonstrated that overexpression of at least one module of the splicing factors SRSF2, SRSF3, and SRSF7 correlated with high CMYC activity across thirty-three cancer types [34].

The transcriptional activity of CMYC indirectly affects other oncogenes and key oncogenic processes by deregulating splicing. Through the overexpression of hnRNPH splicing factor, CMYC affects the RAS/RAF/ERK signaling pathway. A high level of CMYC and hnRNPH correlates with the expression of full-length A-RAF kinase. A-RAF activates oncogenic RAS signaling and inhibits apoptosis by binding to proapoptotic MST2 kinase. In cells with a low level of CMYC and hnRNPH, A-RAF is spliced into a shorter isoform that cannot bind to MST2. A-RAF_short_ still binds to RAS, but in a way that inhibits RAS activation [35]. One of the hallmarks of cancer is the conversion of glucose into energy via aerobic glycolysis instead of oxidative phosphorylation. This process is governed by PKM2, a pyruvate kinase isoform expressed mainly in embryonic and cancer cells, in contrast to the PKM1 isoform present in most adult normal cells. Overactive CMYC upregulates transcription of PTBP1, hnRNPA1, and hnRNPA2 splicing factors, resulting in a high PKM2/PKM1 ratio [36]. Zhang et al. found that another member of the MYC family, NMYC, similarly causes the upregulation of the same splicing factors, PTBP1 and HNRNPA1, leading to the expression of pro-oncogenic PMK2 isoform [37].

**Figure 1:**
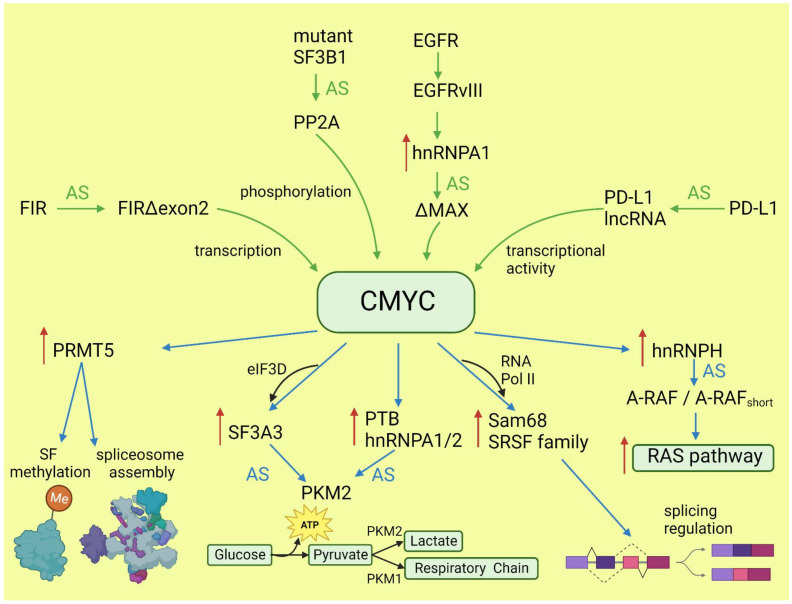
Effects of CMYC on splicing (blue arrows) and the influence of altered splicing on CMYC (green arrows). Red arrows: increased expression; AS: alternative splicing; SF: splicing factor.

CMYC not only initiates transcription but also regulates transcription rate [38]. A pre-mRNA splicing occurs co-transcriptionally and the transcription rate influences the selection of alternative exons [13,39]. CMYC controls the transcription and splicing of a splicing factor, Sam68 pre-mRNA, in prostate cancer by binding to the promoter and increasing the RNA Pol II processivity [40]. Splicing factors (SF) frequently use an autoregulatory loop to control their own level: when SF protein level is high, it induces incorporation of a poison exon that triggers nonsense-mediated decay (NMD) of the transcript. In prostate and breast cancer, it was shown that CMYC not only increased the transcription of SRSF3 (and several other splicing factors) but also prevented incorporation of the poison exon [41]. The exact mechanism remains to be elucidated, but one may hypothesize that the exon skipping is caused by the increased transcription rate induced by CMYC. Selected effects of activated CMYC on splicing are summarized in Figure 1.

### 2.2. RAS and Downstream Pathways

KRAS, together with NRAS and HRAS, belongs to the small GTPases family, which under physiological conditions cycles between active (GTP-bound) and inactive (GDP-bound) conformations in response to stimulation of cell surface receptors such as HER2, EGFR, or CMET. KRAS is commonly mutated in human cancers; however, the percentage of mutations varies between cancer types [42]. Mutations most frequently occur in codons for glycine at amino acid position 12 or 13, causing KRAS hyperactivity that contributes to its transforming properties [43]. Signaling cascades are triggered by the binding of the active RAS to RAS-binding domains within several known RAS effector pathways. The most important, in the cancer context, are the PI3K-AKT and MAPK signaling pathways [44].

**Figure 2:**
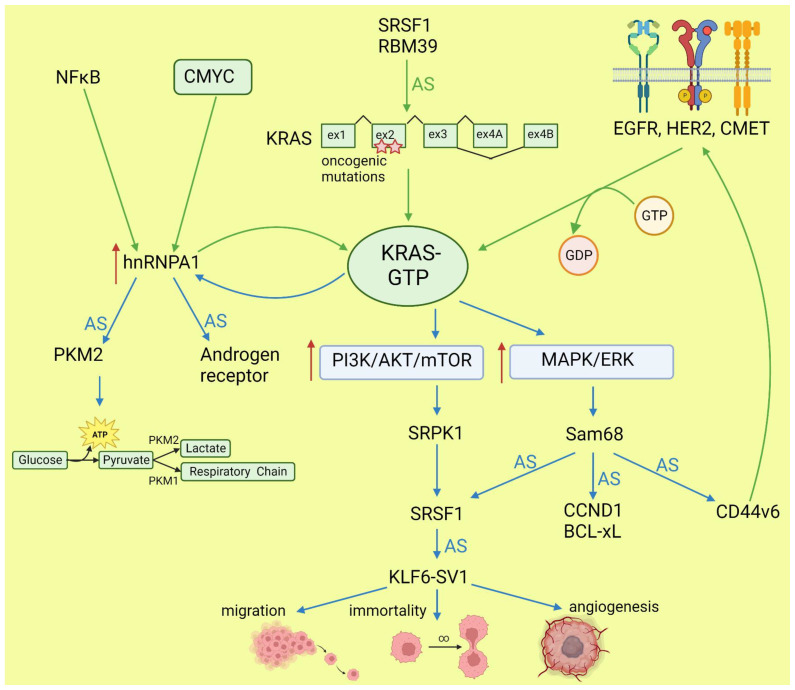
Effects of KRAS on splicing (blue arrows) and influence of altered splicing on KRAS (green arrows). AS: alternative splicing; red arrows: increased expression or pathway activity.

Lo et al. found that in lung cancer cells expressing either WT or mutant KRAS variants, the phosphorylation of SR splicing factors was reduced in those with mutant KRAS expression. This observation correlated with changed cassette exon skipping or inclusion in mutant vs. WT KRAS [45]. Correct phosphorylation of SR proteins by SRPK1 and CLK1 kinases is indispensable for their binding to splicing enhancer sequences on pre-mRNA [46]. It is known that signaling through PI3K/AKT activates SRPK1 kinase [47]; however, the link between RAS and SRPK1 activation was not directly demonstrated. In hepatocellular carcinoma, activation of the RAS/PI3K/AKT pathway leads to the expression of KLF6 splicing variant 1 (KLF6-SV1), which antagonizes the function of full-length KLF6 [48]. This protein is a tumor suppressor. The sorter isoform is an oncogene found in many tumors and involved in proliferation, metastasis, and angiogenesis [49]. KLF6 splicing is regulated by SRSF1 splicing factor [50]. Cheng et al. found a positive feedback loop involving activated RAS and an alternative isoform of CD44 (CD44v6) that can be a coreceptor of growth factor receptors. Activation of the RAS/MAPK/ERK pathway correlated with a preference for exon v6 inclusion. CD44v6, in turn, sustained signaling through tyrosine receptor kinases which activate RAS and downstream pathways, boosting cancer cell proliferation. The author suggested that this positive feedback loop could be a mechanism of the RAS-dependent pathway’s activation in cancers without oncogenic RAS mutations [51]. Alternative splicing of CD44 is regulated by a splicing factor, Sam68, which is phosphorylated by ERK, an effector of the RAS/MAPK/ERK pathway [52]. The same signaling pathway may be responsible for alternative pro-oncogenic splicing of other Sam68-dependent genes, namely, CCND1, SRSF1, BCL-xL, and mTOR [53]. Notably, mTOR is a key effector part of the PI3K/AKT/mTOR pathway that can be activated by RAS [44]. The effects of mutated KRAS on splicing are summarized in Figure 2.

### 2.3. Mutant p53

The *TP53* gene is a tumor suppressor, while its hotspot mutants gain oncogenic activity (named gain of function, GOF) and are involved in all hallmarks of cancer [54]. In contrast to wild-type proteins, p53 GOF mutants do not bind directly to promoters, but interact with different transcription factors to control expression of target genes [55]. In addition to transcription control, p53 mutants contribute to oncogenesis via interaction with TAp63 and TAp73 [56] to inhibit their proapoptotic activity, and with ID4 in angiogenesis stimulation [57,58].

While many researchers have studied the alternative splicing of the *TP53* transcript and demonstrated the role of alternative isoforms in cancer development (discussed in more detail later), little is known about the role of mutant p53 in alternative splicing regulation. Escobar-Hoyos et al. demonstrated that in pancreatic cancer, mutant p53 upregulates the expression of hnRNPK splicing factor. This leads to the mis-splicing of GAP proteins and activates the oncogenic RAS pathway [59]. In addition, the RNA-Seq results underlying this study indicate other genes alternatively spliced under the influence of the p53 mutant. Pruszko et al. found, in breast cancer, a ribonucleoprotein complex composed of mutant p53, a splicing factor SRSF1, ID4, and lncRNA MALAT1. The complex altered a splicing of the VEGFA transcript, thus promoting proangiogenic isoforms over antiangiogenic isoforms [58]. A proangiogenic role of the studied complex was confirmed in vivo in a zebrafish model [60]. These two independent studies indicated that GOF p53 mutants influence alternative splicing in cancer. However, published results are limited to two cancer types and focused on a few target genes. Further studies based on a broad spectrum of tumors are needed to understand the role and interactions with other oncogenes of the GOF p53 mutants in alternative splicing regulation.

## 3. Driver Oncogenes Regulated by Alternative Splicing

### 3.1. TP53 Regulation by Splicing

**Figure 3:**
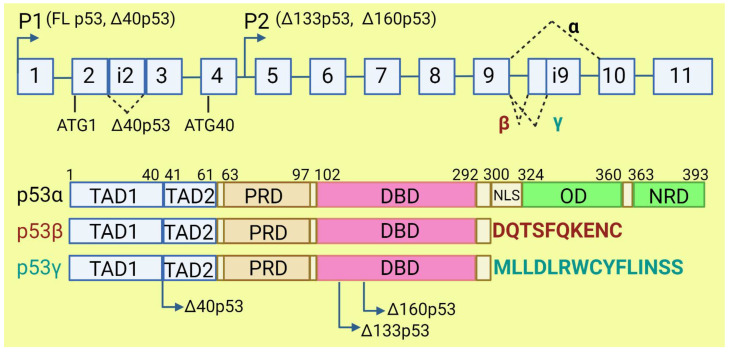
The human *TP53* gene and p53 isoforms. (**Upper panel**): schematic representation of *TP53* gene. Exons are numbered from 1 to 11. Alternative splicing of introns i2 and i9 provides alternative p53 isoforms. P1 and P2: alternative promoters. ATG1 and ATG40: alternative transcription start sites. (**Lower panel**): representation of p53 domains. TAD: transactivation domain; PRD: proline rich domain; DBD: DNA binding domain; NLS: nuclear localization signal; OD: oligomerization domain; NRD: negative regulation domain. Isoforms β and γ lack C-terminal domains, including OD, replaced by DQTSFQKENC and MLLDLRWCYFLINSS, respectively. Isoforms Δ40p53, Δ133p53, and Δ160p53 have a deletion from N-terminus.

The *TP53* gene produces at least 12 isoforms with different features that might prevent or promote cancer development. The canonical, full-length p53 (FLp53) protein has seven functional domains: N-terminal transactivation domains TAD1 and TAD2, a proline-rich domain, a DNA-binding domain, a nuclear localization signal, an oligomerization domain, and a negative-regulation domain [61]. The α (full-length), β, and γ variants of p53 result from alternative splicing of the C-terminus. β and γ isoforms are formed by partial retention of intron 9 (i9). The resulting alternative exons, 9b or 9g, contain a stop codon, leading to the replacement of the oligomerization domain into 10 amino acids (DQTSFQKENC) in p53β and 15 amino acids (MLLDLRWCYFLINSS) in p53γ (Figure 3). SRSF1 and SRSF3 regulate the splicing of i9 and favor the expression of FLp53 [62,63]. Marcel et al. observed that inhibiting CLK kinase or silencing SRSF1 upregulated the expression of p53β and p53γ isoforms in the breast cancer cell line MCF7 [63]. In contrast to SRSF1 and SRSF3, SRSF7 has been reported to enhance p53β expression in response to ionizing radiation [64]. Each of the α, β, and γ isoforms may also be changed from the N-terminus through alternative splicing of intron 2 (Δ40), alternative start of translation from internal IRES (Δ40, Δ160), or transcription from internal promoter (Δ133, Δ160) [65,66] (Figure 3). The co-expression of so many isoforms and the difficulty of distinguishing between them using available molecular biology methods make it challenging to evaluate their role in cancer.

The p53β and p53γ isoforms, often described as antioncogenic, may have important implications for cancer prognosis and therapy [67]. Bourdon et al. observed that breast cancer patients who co-expressed mutant p53 and p53γ had much better prognosis, lower recurrence rate, and longer overall survival than those who had only mutant p53 expression [68]. It would be worthwhile studying if high levels of SRSF1, frequently observed in cancers, contribute to the GOF of p53 mutants by preventing expression of p53β and p53γ isoforms. These isoforms retain the ability to induce the expression of wild-type p53-dependent genes. P53β increases the transcriptional activity of p53α on p21 and BAX promoters, while p53γ only increases its activity on BAX promoters [63]. However, p53β poorly activates MDM2 promoters, which may decrease p53 degradation and contribute to cell cycle arrest and apoptosis. Due to the lack of tetramerization domain, p53β and p53γ cannot form a complex with full-length p53, but they precipitate together when bound to a p53-responsive element on the promoter [63].

The overexpression of Δ40p53 isoform was observed in different neoplasia: in breast cancer and acute lymphocytic leukemia it was correlated with worse clinical outcome [69,70], while in melanoma and ovarian cancer it was connected with better prognoses [71,72]. Further, in vitro studies in cancer cell lines provided contradictory observations [65]. This discrepancy may be related to the proportion between Δ40p53, FLp53, and other p53 isoforms, on the one hand, and mutations in the *TP53* gene, on the other, but further studies are needed before Δ40p53 can be used as a prognostic biomarker [65,73]. Generally, higher expression of Δ40p53α rather inhibits p53′s suppressive activity, but equal or lower expression might support p53 features. The effect of Δ40p53α on FLp53 activity might also be cell-specific [73]. All Δ40p53 isoforms lack part of the N-terminal transactivation domain, TADI, but retain the second part, TADII. Therefore, Δ40p53 in a complex with FLp53 can prevent expression of TADI-dependent genes but retains the ability to activate TADII-dependent genes in a complex or independently from FLp53. The Δ40p53-FLp53 complex binds to target genes in the form of heterotetramer (a dimer of dimers) [74]. Δ40p53, independently from FLp53, regulates expression of antiapoptotic ligand netrin-1 and its receptor, UNC5B. Netrin-1 is overexpressed in several aggressive cancers, such as melanoma, colorectal cancer, and breast cancer, and its expression correlates with the expression of Δ40p53 [75]. Δ40p53α lacks a binding site for MDM2 and lacks major activating phosphorylation sites. Δ40p53 may reduce FLp53 degradation since heterotetramers which contain Δ40p53 have disturbed binding to MDM2 [73]. High expression of Δ40p53 is observed in embryonic tissues, where it contributes to the pluripotency, proliferation, and migration of the cells [76], suggesting that a similar role may be played in cancer stem cells, which requires further research.

The Δ133p53 isoform lacks the transactivation domain, the proline-rich domain, and part of the DNA binding domain [61]. It can form heterotetramers with FLp53 and other p53 isoforms which contain the oligomerization domain. Therefore, Δ133p53 affects the transcriptional activity of FLp53. Many scientific reports indicate that Δ133p53 does not cause malignant transformation [66,77], but it may be important for the progression of benign to aggressive tumors [78,79]. Δ133p53 is overexpressed in gastric, colon, lung, and breast cancers, and in melanoma [79,80,81,82]. The overexpressed Δ133p53 isoform contributes to cancer invasiveness by upregulating the JAK-STAT and RhoA-ROCK signaling pathways. Interleukin-6 (IL-6) contributes to proinflammatory and oncogenic phenotype and acts as mediator of this process [83]. In contrast to FLp53, Δ133p53 inhibits antiangiogenic factors, such as interleukin 12A and matrix metallopeptidase 2, and upregulates proangiogenic factors such as angiogenin, midkine, hepatocyte growth factor, and angiopoietin-like 4. However, silencing of p53 has no impact on the expression of these genes. The ratio of p53 to Δ133p53 may influence the expression of anti- and proangiogenic factors. It was indicated that all isoform variants of Δ133p53 except Δ133p53β stimulate angiogenesis in tumors [84]. Overexpression of Δ133p53 is also linked to upregulation of MDM2, which might influence p53 degradation [85]. Δ133p53 isoform is able to inhibit p53-mediated apoptosis and G1 cell cycle arrest without inhibiting p53-mediated G2 cell cycle arrest in response to doxorubicin treatment. This is possible due to the downregulation of p21 and upregulation of antiapoptotic Bcl-2 by this isoform. Downregulation of Δ133p53 leads to upregulation of proapoptotic protein NOXA. Silencing of Δ133p53 also elicits an increase in caspase 3 cleavage [85]. Upregulation of Δ133p53 with simultaneous downregulation of p53β leads to the inhibition of p53-mediated replicative senescence in human fibroblasts. Overexpression of Δ133p53 with concomitant downregulation of p53β was observed in colon carcinoma, whilst in benign adenoma, the profile of p53 isoform expression was the opposite. This suggested that carcinoma escaped from the senescence barrier. The influence of Δ133p53 on senescence might be caused by altering p53 target genes’ activation, e.g., by a tumor suppressor, miR-34a, which induces apoptosis, senescence, and G1 cell cycle arrest in response to DNA damage [80].

Candeias et al. demonstrated that cancer cells overexpressing Δ160p53 present a phenotype just like cells with GOF p53 mutants: increased survival, proliferation, invasion, and altered tissue architecture. In their research model, expression of full-length mutant p53 without Δ160p53 caused a lack of cancer hallmarks. Knockdown of mutant p53 did not result in restoring apoptosis without knockdown of Δ160p53 [86]. The Δ160p53β isoform was found in U2OS, T47D, and K562 cancer cell lines and treatment with hemin decreased its expression [87]. In melanoma, Δ160p53 isoforms can stimulate proliferation and migration. Treatment with anticancer drugs such as doxorubicin, etoposide, and cisplatin leads to upregulation of Δ160p53 in melanoma cells [88].

In cancer, mutations within the coding sequence are the most studied and best understood. Far fewer studies focus on mutations in introns and their consequences for proper splicing. Several studies indicate that such mutations account for a low percent of all point mutations in *TP53* and can lead to a truncated protein lacking the C-terminal domain [89,90,91]. Splicing mutations in *TP53* may be associated with poorer survival prognosis compared to wild-type p53 [92]; however, they are more difficult to detect and it is more difficult to predict the outcome [93].

GOF p53 mutants are characterized by greater stability compared to the wild-type protein. One mechanism for this phenomenon is related to the alternative splicing of E3 ubiquitin ligase MDM2 [94,95]. In normal cells, MDM2 ubiquitylates and targets p53 for degradation [96]. MDM2 amplification or overexpression is observed in many cancers [97,98]. However, MDM2 does not prevent accumulation of mutant p53 in tumors [99,100]. The most abundant alternative isoform of MDM2 overexpressed in cancers is MDM2-ALT1 (MDM2-B), which lacks a p53-binding domain. The alternative splicing of MDM2 is controlled by SRSF1 and SRSF2 working in an opposing manner: SRSF1 promotes exon 11 skipping and MDM-B expression while SRSF2 prevents exon 11 skipping [101,102]. MDM2 forms dimers and/or oligomers by the RING domain, and this process is necessary for efficient ubiquitination of p53. MDM2-B retains the RING domain and, thus, the ability to bind to MDM2, thereby blocking p53 ubiquitination. Moreover, MDM2-B increases MDM2 cytoplasmic localization and, consequently, decreases its binding to mutant p53 in the nucleus [99]. Aptullahoglu et al. demonstrated that spliceosome inhibition leading to aberrant MDM2 splicing can be used to treat wild-type p53-expressing tumors. They combined E7107 spliceosome inhibitor with RG7388 MDM2 inhibitor to block the E3 ligase on the splicing and protein level, provoking the accumulation of p53 and apoptosis. In addition, inhibition of the spliceosome resulted, by intron retention, in the expression of the p21 isoform, which was unable to inhibit the cell cycle and protect cells from apoptosis [103].

### 3.2. CMYC

Functional alternative isoforms of CMYC are not reported in the literature but alternative splicing of other proteins may affect CMYC activity in cancers. FUSE-binding protein-interacting repressor (FIR) is a suppressor of CMYC transcription. A splice variant of FIR that lacks exon 2 in the transcriptional repressor domain (FIRΔexon2) presents dominant negative activity towards full-length FIR; thus, the expression of FIRΔexon2 upregulates CMYC transcription. The increased ratio of FIRΔexon2/FIR contributes to human colorectal and hepatocellular carcinomas as well as lymphomas [104]. Mutations in *SF3B1*, the gene coding the most commonly mutated spliceosome component across cancers, alters the splicing and promotes the decay of mRNA, coding a specific subunit of the PP2A serine/threonine phosphatase and increasing phosphorylation and, consequently, the stability of CMYC [105]. This observation is particularly valuable, because SF3B1 may be targeted with several inhibitors, and it was demonstrated that patients with overactive MYC are more sensitive to these drugs [29]. In lung adenocarcinoma, under the influence of IFNγ, the protein coding gene PD-L1 may be spliced into long non-coding RNA PL-L1-lnc. This alternative transcript does not encode a protein but is functional. Through direct interaction with CMYC, it boosts its activity as a transcription factor and contributes to the increased proliferation and invasiveness of neoplastic cells [106]. EGFRvIII is a constitutively active variant of EGFR formed by genomic rearrangement. It is highly expressed in glioblastoma multiforme (GBM), where is detected at an overall frequency of 25–64% [107]. EGFRvIII induces a broad change in the alternative splicing program via upregulation of the heterogeneous nuclear ribonucleoprotein A1 splicing factor. HnRNPA1 promotes inclusion of exon 5 in the transcript encoding the CMYC-interacting partner MAX. The resulting shorter isoform ΔMAX enhances CMYC-dependent transformation [108]. The impact of alternative splicing on CMYC is summarized in Figure 1.

### 3.3. KRAS and Downstream Pathways

KRAS may be spliced into two isoforms with alternative exon 4: KRAS4A and KRAS4B. Both isoforms are oncogenic if they have a mutation in G12 or G13 [109]. The two isoforms differ in their C-terminal domain, referred to as the hypervariable region (HVR), which determines the possible post-translational modifications and membrane localization [110]. Although most of the interactome is the same for both isoforms, some proteins interact specifically with one or the other [111,112]. This is related to the distinct association to the membrane and intracellular localization. For example, KRAS4A undergoes palmitoylation–depalmitoylation cycles, unlike KRAS4B. This allows it to bind to heterokinase HK1 on the outer mitochondrial membrane and stimulate HK1 activity, thereby increasing glycolysis in cancer cells [113]. Moreover, KRAS4A binds more strongly to RAF1, augmenting downstream ERK signaling and anchorage-independent growth [112]. The regulation of KRAS splicing in cancers is not clear. Hall et al. reported that in colon cancer with inactive APC, the key role is played by SRSF1 [114]. KRAS4A expression may be also decreased by splicing inhibitor Indisulfam, which targets the protein RBM39 associated with the spliceosome [115] (Figure 2).

Alternative splicing also affects downstream components of RAS/MAPK and PI3K/AKT pathways [116]. A-RAF, B-RAF, and C-RAF can be spliced into dominant-negative isoforms with only an RAS-binding domain or constitutively active, oncogenic isoforms containing only a kinase domain. The splicing of A-RAF in cancer is regulated by hnRNPH, and indirectly by CMYC, which upregulates the level of the splicing factor [35]. In hepatocellular carcinoma, it was demonstrated that hnRNPA2 also causes constitutive activation of the RAS pathway by the splicing of A-RAF [117]. The MKNK2 gene is expressed as two isoforms: MNK2a and MNK2b kinases. The alternative splicing of MKNK2 is regulated by proto-oncogene SRSF1 [118]. While MNK2a can suppress RAS-induced transformation, MNK2b is pro-oncogenic. It phosphorylates the translation initiating factor eIF4E—an effector of RAS-dependent pathways—increasing translation in cancers [119]. In the PI3K-AKT-mTOR pathway, both PI3K and mTOR may be spliced into more active oncogenic isoforms [120,121]. The mTORβ isoform, despite losing most of the protein–protein interaction domains, retains its kinase activity and ability to complex with Rictor and Raptor proteins. Most importantly, mTORβ accelerates proliferation by shortening the G1 phase of the cell cycle, and its overexpression is sufficient for immortal cell transformation [121]. Increased activation of AKT further affects aberrant alternative splicing by phosphorylation of SRPK kinase [47].

### 3.4. EGFR and FGFR

The ErbB family of receptors consists of four transmembrane receptor tyrosine kinases: EGFR (ErbB-1), HER2/neu (ErbB-2), HER3 (ErbB-3), and HER4 (ErbB-4). The binding of a soluble ligand to the external domain of the receptor promotes homo- and heterodimerization, which is essential for the activation of the intracellular tyrosine kinase domain and phosphorylation of the C-terminal tail. Phosphorylation of tyrosine residues allows for activation of downstream pathways including RAS/MAPK, PLCγ1/PKC, PI3K/AKT, and JAK/STAT [122]. In cancers, the activity of receptors—in particular, EGFR and HER2—is increased by gene amplification, mutation of the kinase or extracellular domain, and aberrant splicing contributing to several hallmarks of cancer [123,124].

The EGFR gene consists of 30 exons. Skipping of exons 16–17 generates the full-length receptor. Alternative splicing and proteolytic cleavage result in a formation of three soluble isoforms, namely, sEGFRv2, sEGFRv3, and sEGFRv4 [125,126]. They are devoid of an intracellular and transmembrane domain; thus, they are secreted from cells, where they bind ligands but do not transduce signal to downstream signaling pathways. To date, it is not known how the splicing of these isoforms is regulated. In several cancers, including ovarian, breast, and lung cancer, reduced expression of sEGFR isoforms was observed [127,128,129]. These observations pointed to an antioncogenic role for sEGFR isoforms, as they bind EGFR ligands and restrict signals that enhance proliferation and promote tumor transformation. However, elevated sEGFR expression was found in gastric and cervical cancer and was associated with shorter overall survival [130]. These contradictory observations indicate that both the regulation of expression and the role of sEGFR isoforms may be specific to tumor type. Further studies are needed to clarify these differences.

Piccione et al. identified a new splicing variant of EGFR named mini-LEEK (mLEEK) [131]. They found that the isoform is broadly expressed in different tissues and overexpressed in ovary, skin, or lung cancers. mLEEK lacks the extracytoplasmic, transmembrane, and tyrosine kinase domains (exons 2–22) and localizes in the nucleus. It contributes to the cellular response to endoplasmic reticulum (ER) stress and unfolded protein response (UPR) by the transcriptional regulation of the genes involved in these pathways, e.g., molecular chaperon GRP78/Bip. Thus, mLEEK helps cancer cells to survive and proliferate under stress conditions.

HER2 is a driver oncogene in HER-positive breast cancer and in lung, colon, and gastric cancer. Alternative splicing allows for expression of three isoforms in addition to the full-length protein: d16HER2, Herstatin, and p100. Herstatin and p100 isoforms retain, respectively, an intron 8 or 15, which leads to premature stop codons and expression of truncated proteins with only the extracellular domain [132]. These soluble receptor isoforms have auto-inhibitory properties because they bind HER2 ligands but do not activate intercellular signaling. Similarly, they may reduce the effectiveness of anti-HER2 therapeutic antibodies [133,134]. The isoform d16HER2, obtained by exon 16 skipping, is oncogenic. Its splicing is probably regulated by SRPK1 and SR family splicing factors, including SRSF1, which is frequently overexpressed in breast cancer [135]. D16HER2 forms constantly active homodimers and induces oncogenic transformation in several models [136,137,138]. The receptor activates signaling through MAPK, PI3K/AKT, SCR, and FAK kinases, contributing to cell proliferation, EMT, and stemness [138,139].

The fibroblast growth factor receptor family consists of four members with a tyrosine kinase domain, namely, FGFR1-4, and one member without kinase activity—FGFR5 (FGFRL1). Increased activity of FGFR1-4 receptors was reported in many cancers and is attributed to gene amplification, overexpression, mutations, gene fusions, and increased levels of ligands [140]. Upon ligand binding, FGFRs activate the RAS/MAPK/ERK, PI3K/AKT, and JAK/STAT pathways and contribute to cancer cells’ survival, proliferation, and angiogenic signaling [141]. The family of 4 receptors is responsible for recognition of 23 different ligands from the FGF family. The diversity is provided by both heterodimerization and alternative splicing [142]. The main source of variation in ligand recognition is an alternative splicing of Ig loop III observed in FGFR1-3. IgIII has two variants, marked as IIIb and IIIc, depending on the selection between two mutually exclusive exons: 8 or 9 [124,142]. The splice variants are expressed in a tissue-specific manner. FGFR2IIIb is present mostly in epithelial cells while FGFR2IIIc is present mostly in mesenchymal cells [124]. FGFR2 exon switching from the IIIc to the IIIb isoform occurs during normal organogenesis in mice (mesenchymal–epithelial transition) [143]. The opposite process, a switch from IIIb to IIIc, is linked to epithelial–mesenchymal transition and was described in several advanced tumors [144]. FGFR2IIIb is associated with benign and well-differentiated tumors, adhesion to the extracellular matrix, and angiogenesis, while FGFR2IIIc correlates with aggressiveness and epithelial–mesenchymal transition in various types of cancer including colorectal cancer [145,146]. FGFR1IIIc is overexpressed in several carcinomas, e.g., non-small cell lung cancer or glioblastoma, where it allows for autocrine stimulation by FGF5 [147,148]. Less is known about switching between IIIb and IIIc isoforms of FGFR3. In advanced colorectal cancers, the expression of FGFR3IIIb is downregulated, without the influence on FGFR3IIIc. Ectopic overexpression of IIIb isoform inhibits cell growth, while FGFR3IIIc induces proliferation, survival, and colony formation [149]. Marie Lafitte et al. proposed that in epithelial cells, FGFR3 plays a role of tumor suppressor, but if cancer cells undergo EMT, FGFR3 starts to act as an oncogene [150]. The switch between FGFR1-3 IIIb and IIIc isoforms in cancers is linked with EMT-related signaling and regarded as a hallmark of this process [144]. However, it is not yet clear which comes first—the splicing switch of FGF receptors or the activation of other EMT-related pathways such as the TGFβ pathway or the WNT signaling pathway [142]. The expression of FGFR2IIIb is related to the receptor stimulation by FGF7. A lack of this ligand provokes a switch to the IIIc isoform [151]. Further, FGF1 and FGF2 were found to regulate the switch in FGFR2 and FGFR3 [152]. Little is known about splicing factors involved in the exon selection between IIIb and IIIc isoforms. hnRNPH was found to silence the inclusion of exon 9, therefore promoting IIIb isoform [153]. ESRP1 and ESRP2 are splicing factors which may be a link between FGFRs splicing and EMT [154,155].

A less common splicing variation is the skipping of exon 3 of FGFR1, resulting in expression of FGFR1β that lacks one of three Ig-like loops [156]. Tomlinson et al. found that the levels of α (full-length) and β isoforms are similar in normal tissues, while in bladder cancer, FGFR1β is prevalent. Both receptors are activated by the same ligands and signal through the same pathways, but β isoform has higher affinity to FGF1 and is activated by lower concentration of the ligand, which may provide an advantage to cancer cells and speed up their proliferation [157]. Similarly, in breast cancer, both FGFR1 isoforms were able to transform immortalized normal breast MCF-10A cells, as assessed by 2D and 3D colony formation assays, but only FGFR1β increased cell growth and motility. The FGFR1β/FGFR1α ratio was higher in more aggressive basal-like tumors than in luminal tumors. Interestingly, the antiestrogen receptor drug 4-OHT increased the expression of FGFR1β by decreasing the level of splicing inhibitor PTBP1, which in turn sensitized cells to FGFR1 inhibitor BGJ-398. The combination treatment was very efficient in killing breast cancer cells in vitro [158].

## 4. SR and hnRNP Families of Splicing Factors: The Oncogenes That Regulate and Are Regulated by Alternative Splicing

The selection of alternative splice sites is orchestrated by splicing factors that bind to regulatory sequences in pre-mRNA. Some of them, e.g., SRSF1, SRSF6, or hnRNPA1, play a key role in oncogenic transformation [33,159,160]; thus, we discuss them as examples of driver oncogenes and as the main players clearly in control of alternative splicing. The effects of other oncogenes on alternative splicing are usually mediated by splicing factors, as described in previous paragraphs and shown in Figure 1 and Figure 2.

The SR family of splicing factors is characterized by the arginine/serine (RS)-rich domain and at least one RNA recognition motif (RRM) domain [161]. A common manner in which SR family splicing factors autoregulate is through alternative splicing coupled with nonsense-mediated decay (AS-NMD). SR family members possess highly conserved non-coding fragments in their sequence, either as cassette poison exons (PE) or retained introns in 3′ UTR, which—if spliced in—create a premature STOP codon and trigger RNA degradation. Usually, splicing factors bind to their own PE or sequences in 3′UTR and promote its inclusion, thus downregulating their own expression [15]. In cancer patients, alternative splicing of splicing factors is commonly deregulated, contributing to the aberrant splicing pattern in tumors [162]. Interestingly, alternative splicing of 3′UTR is a recently described phenomenon, not limited to splicing factors. It is upregulated in cancers and exceptionally frequent in oncogenes including hnRNPA2B1 and hnRNPA1 [163]. Leclair et al. found that poison exons are differentially included during tumorigenesis and cell differentiation. They demonstrated that SR family splicing factors form a cross-regulatory network to control the levels of other family members in a coordinated manner. Regulation of poison exon inclusion with ASO may be a promising therapeutic strategy, as they demonstrated in breast cancer cell lines [164].

An established oncogene, and the most studied representative of the SR family, SRSF1, has appeared in many previous paragraphs. Another member of the SR family, SRSF6, has emerged in recent years as a potent oncogene whose overexpression translates into stimulation of metastasis, drug resistance, or insensitivity to apoptosis [165]. SRSF6, as well as SRSF4 and TRA2β, can induce transformation of MCF-10A cells, stimulating their proliferation and invasiveness [166]. SRSF6 contributes to apoptosis resistance by splicing regulation of proteins BIM and MNK2. Knockdown of SRSF6 or mutation of its binding site results in an increased level of the most proapoptotic isoform, BimS, in neuroblastoma cells [167]. However, in melanoma cells treated with B-RAF(V600E) inhibitor PLX4720, SRSF6 works the other way around, increasing splicing of BimS [168]. This suggests that SRSF6 regulation of BIM splicing depends on the cell type. In colon and lung cancer, *SRSF6* gene amplification and overexpression correlates with an increased level of antiapoptotic MNK2b isoform and a decrease in proapoptotic MNK2a [169]. SRSF6 promotes migration and invasion, for example, by splicing the ZO-1 gene involved in the cell adhesion process [170]. SRSF6 may stimulate or diminish EMT, depending on the cancer type: in colorectal cancer, downregulation of long non-coding RNA LINCO1133 by TFGβ leads to an increased pool of free SRSF6 and stimulation of EMT [171]; in pancreatic cancer, SRSF6 inhibits this process, while increased expression of miR-193a-5p reduces SRSF6 levels, enabling EMT [172]. In gastric cancer, SRSF6 contributes to chemoresistance by the production of a long isoform of PICALM protein [173]. Individual splicing factors often compete with each other for binding to the same pre-mRNA molecules and oppositely regulate the inclusion/skipping of a given exon. In such a situation, the relative levels of the two proteins are important. E.g., SRSF6 and SRSF1 oppositely affect the expression of VEGFA isoforms and, thus, of angiogenesis: while SRSF1 increases the expression of proangiogenic VEGFAxxx isoforms, SRSF6 promotes antiangiogenic VEGFAxxxb isoforms [174]. The activity of SRSF6, similarly to other SR proteins, is regulated by phosphorylation of the RS domain. IQGAP1 (IQ motif-containing GTPase-activating protein 1) that scaffolds different signaling pathways important in oncogenesis (including RAS and PI3K) and involving many kinases, mediates phosphorylation of SRSF6 and other splicing factors in head and neck cancer cells as well as in normal keratinocytes [175].

Heterogeneous nuclear ribonucleoprotein A1 (hnRNPA1) is a member of a family of RNA-binding proteins that are involved in pre-mRNA splicing, metabolism, and transport. hnRNPA1 is the most abundant member of the family and significantly contributes to malignant transformation [159]. Silencing of hnRNPA1 induces senescence in normal and cancer cells and inhibits cancer-related phenotypes such as colony formation and cell migration [176]. Several studies demonstrated that hnRNPA1 overexpression particularly enhances KRAS activity. The promoter of *KRAS* proto-oncogene contains G-quadruplex structure, which is bound and unwound by several proteins, including hnRNPA1, to enable *KRAS* transcription [177]. KRAS signaling, in turn, increases expression of *hnRNPA1*, creating a positive feedback loop [178] (Figure 2). Several studies have also shown that the *hnRNPA1* gene is a target of activated CMYC in various cancers [36,179,180]. Overexpressed hnRNPA1 represses the splicing of PKM1 and promotes the expression of PKM2 isoform to facilitate the switch of cancer cells to anaerobic glycolysis [36,181]. This effect was recreated in a transgenic zebrafish model overexpressing CMYC and xmrk (activated EGFR homolog) [182]. In castration-resistant prostate cancer, hnRNPA1—upregulated by NFkB and CMYC—leads to the aberrant splicing and expression of constitutively active androgen receptor [179].

## 5. Therapeutic Implications

Despite multiple attempts, key driver oncogenes are still difficult to target therapeutically directly. PRIMA-1 and its derivative PRIMA-1^Met^ (also named APR246) are small molecules that have been designed to restore the ability of mutant p53 to bind and activate wild-type p53-dependent promoters and induce the expression of proapoptotic genes [183]. PRIMA-1^Met^ was tested in clinical studies, alone [184] and in combinations [19], but, despite promising results, is still not included in standard therapeutic protocols. A recent study by Guiley et al. describes covalent compounds that restore the thermal stability of p53 somatic mutant Y220C by interaction with a cysteine residue. This allows the transcription factor to achieve WT-like conformation and activate WT p53 target genes [185]. Inhibitors directly targeting KRAS are effective toward specific mutants; e.g., MRTX1133 [186] targets only G12D mutants, while sotorasib inhibits KRAS-G12C [187]. None of the known inhibitors binds KRAS with hotspot mutations in codon 13 [20]. Two years ago, sotorasib was approved by the FDA for non-small cell lung cancer therapy [188]. Unfortunately, many patients present intrinsic or acquired resistance to this drug [189]. In addition, amplified/overexpressed CMYC creates a problem because this protein does not possess an active side or a pocket which would be a natural target for small molecules. The only molecule demonstrated to inhibit CMYC activity by disrupting its interactions with other proteins is a peptide, Omomyc [190]. Last year, results of a phase I clinical trial presented at a conference showed a good safety profile and some activity justifying further investigation [21]. The therapeutic targeting of driver oncogenes was revised in greater detail by Grześ et al. [27].

The alternative to direct targeting of driver oncogenes is targeting dependent pathways or processes that lead to their increased activity. In this review, we have shown how selected oncogenes interfere with alternative splicing as well as how splicing increases the activity of driver oncogenes. Therefore, there is a growing interest in finding therapies that target alternative splicing in cancer. All approaches can be divided into two groups: broad-spectrum splicing inhibition and specific correction of single splicing events.

### 5.1. Splicing Inhibitors

The literature describes many molecules of natural origin and their derivatives that inhibit the spliceosome and, more specifically, the SF3B1 protein, which is often mutated in cancers, leading to a wide spectrum of changes in the splicing profile. Examples of SF3B1 inhibitors are FR901464, spliceostatin A, pladienolide B, FD-895, sudemycin E, or H3B-8800 [191,192,193]. SF3B1 is responsible for the branch point sequence and 3′splice site recognition [194]. Its mutation leads mostly to an aberrant selection of the 3′ splice side, which in about 50% of cases, results in NMD of the misspliced transcript and, in the remaining 50% of cases, to the expression of mutated protein [195]. It was observed that these inhibitors are more effective towards tumors with mutant SF3B1 [196,197]. However, they are not mutant-specific, which can cause side effects from drugs that were tested in clinical trials [198]. Interestingly, inhibition of SF3B1 with pladienolide B induced, in glioblastoma and pancreatic cancer cell lines, the expression of proapoptotic isoforms BCL-XS and Δ133p53 [199,200]. E7107 and H3B-8800 inhibitors synergize with common chemotherapeutics and CHEK2 inhibitor BML277 in T-cell leukemia therapy [201]. The biflanoid isoginkgetin, isolated from *G. biloba*, prevents recruitment of the U4/U5/U6 tri-snRNP, preventing complex B formation [202]. The emerging target is a protein arginine methyltransferase 5 (PRMT5), upregulated by CMYC, as mentioned earlier [30,203]. Several inhibitors of PRMT5 were tested in vitro and some of them (e.g., GSK3326595, JNJ-64619178, or PF-06939999) were also tested in clinical trials [204]. In leukemias, mutations of spliceosome components are more common than in solid tumors, making them more sensitive to PRMT5 inhibitors [205]. Patients may benefit more from the combination of PRMT5-targeting drugs with PARP or ATR inhibitors [206,207]. Several research groups observed that cancers with overexpressed CMYC are particularly sensitive to drugs inhibiting spliceosome or kinases involved in alternative splicing [29,105,208]. Presumably, the co-occurrence of splicing abnormalities caused by CMYC overexpression with the action of spliceosome inhibitors or splicing factors produces a lethal effect in cancer cells. Thus, patients with CMYC overexpression could potentially benefit most from spliceosome targeting therapy, but this needs to be tested in clinical trials.

To date, there are no inhibitors of regulatory splicing factors. Denichenko et al. tackled the problem in a different manner, designing a decoy RNA oligonucleotide which binds to its respective splicing factors. Anti-SRSF1, RBFOX1/2, and PTB1 oligonucleotides were active both in vitro and in vivo [209]. A similar approach can be used against any overexpressed or overactive splicing factor. Many oncogenic signaling pathways, including PI3K/AKT, MAPK/ERK, and Wnt/βcatenin, contribute to splicing deregulation via activation of SRPK1 and CLK1 kinases [210]. For this reason, their inhibitors have been tested in many cancers, singly and in combinations with other drugs, including EGFR pathway-targeting drugs [211,212,213,214]. The most effective was a combination of SRPIN340 with AKT inhibitor GSK690693 [211]. Iwai et al. identified a novel CLK inhibitor, T-025, which was particularly efficient towards CMYC-driven breast cancer [208]. The natural compound quercetin was found to decrease the expression of hnRNPA1 splicing factor involved in the splicing of the androgen receptor (AR) and the generation of constitutively active isoform AR-V7. Downregulation of hnRNPA1 with quercetine restored the sensitivity of prostate cancer cells to enzalutamide treatment [215].

### 5.2. Targeting of Specific Isoforms

Alternative isoforms of many genes are expressed in a specific cell type at a certain developmental stage. Some isoforms have antioncogenic properties, while others confer an advantage to tumor cells. Examples include PKM1/2, VEGFA165a/b, and MNK2a/b. By binding to regulatory sequences, specially designed modified antisense oligonucleotides can target splicing and induce the skipping of unwanted exons and the expression of desired isoforms. This strategy was successfully tested on various genes such as MNK2a/b [118] and PKM1/2 [216]. Splice switch oligonucleotides (SSOs) have also been designed to destroy and inactivate the ERG gene, which is a driver oncogene in prostate cancer activated by fusion with androgen-dependent TMPRSS2 promoter [217]. Although no SSO has been approved or tested in clinical trials for cancer therapy yet, such drugs have been approved for Duchenne muscular dystrophy (Eteplirsen) and spinal muscular atrophy (Nusinersen) [218,219], which confirms the feasibility of this kind of therapy in humans. SSO chemistry, mechanism of action, and application in cancer were recently revised in detail elsewhere [220,221]. A common challenge for all oligonucleotide-based therapies is the efficient delivery of DNA or RNA molecules to the target cells [222]. Soudah and colleagues developed CLIP6-PNA-peptide conjugates, which are peptide nucleic acid conjugated to a cell-penetrating peptide. They showed that their splice-switching oligonucleotides could enter human glioblastoma cells via a non-endosomal mechanism and modulate the expression of MNK2a/b isoforms [223]. Scharner et al. used receptor-mediated uptake of SSOs via tissue-specific receptors to target cells derived from a specific tissue. They used triantennary N-acetylgalactosamine (GalNAc) (GN3)-conjugated antisense oligonucleotides (ASO) that bind to the asialoglycoprotein receptor (ASGP-R) exclusively expressed on hepatocytes [224].

Alternative isoforms of genes associated with certain hallmarks of cancer can be used as a tool to screen for new drugs. For example, Li et al. used a FGFR2-based splicing reporter as an EMT marker and searched for a compound that could inhibit this process [225]. Similarly, a VEGFA-based splicing reporter was used to identify compounds that could promote the expression of the antiangiogenic VEGFA165b isoform [226].

### 5.3. Resistance to Targeted Therapies

Another aspect to consider while discussing therapy and alternative splicing is the emergence of alternative isoforms that confer resistance to targeted therapies. Vemurafenib is a drug that inhibits the mutated BRAF-V600E kinase, which is a driver oncogene in melanoma. However, many patients develop resistance due to the expression of BRAF isoforms that lack the RAS-binding domain, required for the proper regulation of BRAF activity [227,228]. Prostate and breast cancers often depend on hormone receptors: an androgen receptor (AR) and an estrogen receptor (ER), respectively. These receptors are targeted therapeutically by inhibitors such as abiraterone and enzalutamide (anti-AR) or tamoxifen (anti-ER). The constitutively active isoforms AR-V7, AR-v567es in prostate cancer [229,230], and ERα36 in breast cancer [231], activate target genes in a ligand-independent manner and are insensitive to inhibitors. Similarly, aberrant splicing of some tumor suppressors contributes to therapy resistance. For example, the BRCA1Δ11q isoform confers resistance to PARP inhibition and cisplatin [232]. The mis-splicing of the proapoptotic protein BIM produces an isoform that lacks the BH3 domain, which is necessary to induce apoptosis, leading to resistance to tyrosine kinase inhibitors [233,234]. Alternative splicing of CD19 and CD22 in B cell acute lymphoblastic leukemia results in acquired resistance to CAR-T-based therapy due to loss of epitopes recognized by CAR-T lymphocytes [235,236]. It is not clear how d16HER2—the shorter isoform of the HER2 receptor discussed in one of the previous chapters—contributes to trastuzumab treatment efficacy. Some researchers observe that d16HER2-expressing patients respond better to the drug [139], while others claim that this isoform confers trastuzumab resistance [237].

## 6. Conclusions

In this work, we focused on presenting the interaction between leading oncogenes and alternative splicing. On the one hand, oncogenes such as CMYC, KRAS, and mutant p53 affect alternative splicing; on the other hand, their activity can be increased by this process. Many reviews discussed how the selected oncogene affects the hallmarks of cancer: CMYC [238], KRAS [44], mutant p53 [54], SRPK1 [9]. Here we emphasized that none of these oncogenic proteins and pathways works alone, but, rather, cooperate to contribute to hallmarks of cancer.

Therapeutic targeting of driver oncogenes or splicing machinery faces multiple problems. Some drugs reached clinical trials but were tested as single agents and did not present an advantage over standard protocols. A better understanding of the relationships between splicing and other common oncogenic pathways will allow the designing of combined therapies and profit from the synergistic effect of simultaneous inhibition of interrelated molecular processes.
